# Transcriptome Analysis of Hormone-and Cuticle-Related Genes in the Development Process of Deutonymph in *Tetranychus urticae*

**DOI:** 10.3390/insects12080736

**Published:** 2021-08-17

**Authors:** Gang Li, Xinyao Gu, Shunhua Gui, Jianjun Guo, Tianci Yi, Daochao Jin

**Affiliations:** Guizhou Provincial Key Laboratory for Agricultural Pest Management of the Mountainous Region, Institute of Entomology, Scientific Observing and Experimental Station of Crop Pest in Guiyang, Ministry of Agriculture, Guizhou University, Guiyang 550025, China; agrgli@163.com (G.L.); agr.xyku18@gzu.edu.cn (X.G.); shgui3@gzu.edu.cn (S.G.); jjguo@gzu.edu.cn (J.G.); tcyi@gzu.edu.cn (T.Y.)

**Keywords:** *Tetranychus urticae*, development process, transcriptome, hormone, cuticle

## Abstract

**Simple Summary:**

The two-spotted spider mite, *Tetranychus urticae*, is an important agricultural pest. Gene expression dynamics during different development time points of deutonymphal development process in *T. urticae* were investigated. The transcriptome sequencing aims to identify hormone- and cuticle-related genes that regulated the development process of spider mite. Moreover, this comprehensive transcriptome will provide a foundation for further research of development process in *T. urticae*.

**Abstract:**

*Tetranychus urticae* is an important agricultural pest that feeds on more than 1100 plant species. To investigate gene expression network in development process of deutonymph, a comprehensive transcriptome analysis of different developmental time points of deutonymph in *T. urticae* was performed. Comparing with expression profile of 7 h, 309, 876, 2736, and 3432 differential expression genes were detected at time points 14 h, 21 h, 28 h, and 35 h, respectively. The expression dynamic analysis indicated that genes in hormone- (ecdysteroid and juvenile hormone) and cuticle- (chitin and cuticle proteins) related pathways were indispensable for development process in deutonymph. Among hormone related pathway genes, the ecdysteroid biosynthesis pathway genes were highly expressed at the growth period of development process, which is opposite to the expression patterns of juvenile hormone biosynthesis pathway genes. For cuticle related pathway genes, 13 chitinase genes were identified in the genome of *T. urticae*, and 8 chitinase genes were highly expressed in different time points of developmental process in the deutonymph of *T. urticae*. Additionally, 59 cuticle protein genes were identified from genome, and most of the cuticle protein genes were expressed in the molting period of developmental process in deutonymph. This study reveals critical genes involved in the development process of deutonymph and also provides comprehensive development transcriptome information for finding more molecular targets to control this pest.

## 1. Introduction

The epidermis of arthropods is not only the shield to protect themselves from damage in the environment and the loss of water for better adapting to the environment, but also the hard exoskeleton on the insect body to provide strongly mechanical support and maintenance of morphology [1]. The insect cuticle is composed of chitin and cuticle proteins (CPs), which bind with each other to keep stable of cuticle structure to maintain the insect physical function [2]. Chitin is a polysaccharide of widespread existence in organisms [3]. In insects, chitin is primarily distributed in ectodermal epithelial tissues, such as cuticles, gut, and trachea [4]. Chitin is synthesized by a series of enzymes, for example, chitin synthases [5,6,7]. Insects need to degrade the old cuticle for the periodical molting during the developmental process. Insect chitinase is required for chitin degradation in the old cuticle, belonging to the glycoside hydrolases 18 family (GH18) [8]. Chitinases display different expression dynamics in different development process and tissues of insects [9]. Further, the cuticle protein is an important component of insect cuticle layers, recognized by the conserved structure the Rebers and Riddiford Consensus (R&R Consensus) containing the chitin binding domain [10]. The CPR protein family is the largest group of the cuticle proteins, which is generally divided into three subfamilies, RR-1, RR-2, and RR-3 that are related to different types or regions of the cuticle [11]. In these different types of CPR proteins, the RR-1 type proteins are related with the flexible cuticles, while RR-2 family proteins are involved in the rigid cuticles [11,12].

However, insect exoskeleton restricts its own development, and needs to periodically shed the old cuticle while forming a new cuticle. This process is collectively regulated by endocrine hormones such as ecdysone and juvenile hormone to finish the development of physiological processes, such as external morphology and internal organs. The dynamic changes of two hormones regulate developmental transition from developmental period to molting period: a high level of juvenile hormone could inhibit the metamorphosis and induce the larval–larval molting during the larva instars, while the absence of juvenile hormone causes the metamorphosis triggered by ecdysone [13].

Spider mites are important agricultural pests because of their multiple host plants, quick resistance development and short lifecycle (two weeks). The function of chitin related genes and the expression patterns of ecdysteroid and juvenile hormone pathway genes in the molting process of spider mites have been reported in previous studies [14,15,16], but there is no study about the regulatory networks in development process in spider mites. To better understand the regulatory networks of development process in mites, we used the two-spotted spider mite *Tetranychus urticae* for further transcriptome analysis. The mite *T. urticae*, a worldwide agricultural pest that often invade greenhouses, is a model mite and could feed on more than 1100 plant species. In present study, we performed a comprehensive transcriptome analysis of different development process of deutonymph in *T. urticae* and aimed to investigate the expression networks in developmental process. This result will lay a foundation for the gene screening in the development process of mite.

## 2. Materials and Methods

### 2.1. Mite Culture

*Tetranychus urticae* adults were collected from the pea bean leaves in Guiyang, Guizhou Province, China. The mites were fed on soybean seedlings at 27 ± 1 °C, 60 ± 10% relative humidity, and 14 h:10 h (L:D) photoperiod in the laboratory. Motionless protonymphs were prepared for different developmental time points of deutonymphs collection. Five hundred deutonymphs in 7 h, 14 h, 21 h, 28 h and 35 h were collected for transcriptome sequencing. Three biological replicates were set in each time point.

### 2.2. RNA Isolation, and Transcriptome Sequencing

RNA of all samples was extracted by the TRIzol (Invitrogen, Carlsbad, CA, USA). RNA concentration and integrity were assessed using NanoDrop 2000 (Thermo Fisher Scientific, Wilmington, DE, USA) and Agilent Bioanalyzer 2100 system (Agilent Technologies, CA, USA). 1 μg RNA was reversed to cDNA by random hexamer primer. Then, the library was constructed by Illumina platform.

### 2.3. Analysis of RNA-Seq Data

The row data was removed the adaptor, poly-N and low quality reads to obtain clean data. At the same time, Q30 and GC content of the clean data were calculated. The clean data was then mapped to the reference genome of *T. urticae* by the soft Hisat2 [17]. The genes function was annotated based on the following databases, including NR, GO, Pfam, COG, KOG, and KEGG.

### 2.4. Differential Expression Analysis

The gene expression levels were calculated by the fragment per kilobase of exon per million fragments mapped (FPKMs). The differential expression analysis of genes in different time points was performed by the soft DESeq2 using the negative binomial distribution. The *p*-values were analyzed by the Benjamini and Hochberg’s approach. Genes with fold change ≥ 2.0 and *p*-value < 0.01 was assigned as significantly differential expression. The GO and KEGG enrichment analysis of differential expression genes were performed by the GOseq Rpackages and KOBAS software, respectively.

### 2.5. Expression Dynamics Analysis of Differential Expression Genes in Development Process

The expression dynamics of differential expression genes in different time points of development process were analyzed by the software Short Time-series Expression Miner (STEM, version 1.311). A total of 20 expression clusters were set to generalize all differential expression genes in development process of deutonymph. The expressions of differential expression genes were normalized by log_2_(7 h/7 h), log_2_(14 h/7 h), log_2_(21 h/7 h), log_2_(28 h/7 h), and log_2_(35 h/7 h). The cluster with *p* < 0.05 was identified as significant development expression cluster.

## 3. Results

### 3.1. RNA-Seq Data

The development process of deutonymph in *T. urticae* was divided to two periods: period I (7 h–21 h, growth stage), and period II (28 h–35 h, molting stage). To identify the genes related with development process of spider mite systematically, 15 RNA libraries in different development time points (7 h, 14 h, 21 h, 28 h, and 35 h) with three biological replications for each development time point were constructed by Illumina Hiseq platform. After filtering low-quality reads, 92.59 Gb clean data was obtained from 15 samples. The clean data was submitted to the NCBI SRA database with the BioProject accession number of PRJNA752938. The number of clean reads and clean bases from 15 samples was from 19,239,246 to 23,280,649 and 5,753,690,284 to 6,910,020,364, respectively. The Q30 was evaluated more than 92.34%. The GC content of clean reads from all samples ranged from 37.49% to 39.22% (Table 1). Additionally, the clean reads were aligned with the *T. urticae* genome (https://bioinformatics.psb.ugent.be/orcae/overview/Tetur (accessed on 6 August 2021)). The mapped rate of 15 samples ranged from 85.26% to 91.27%. In addition, 978 genes were not annotated to the genome of *T. urticae* and viewed as novel genes.

### 3.2. Differential Expression Genes Analysis in Development Process of Deutonymph in T. urticae

**Figure 1:**
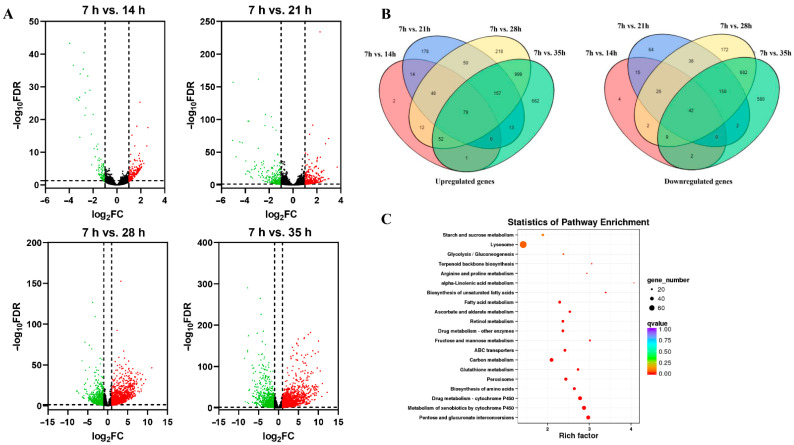
(**A**) Volcano plots of differential expression genes in different developmental time points (14 h vs. 7 h, 21 h vs. 7 h, 28 h vs. 7 h and 35 h vs. 7 h) of deutonymph in T. urticae. (**B**) The venn diagram of the numbers of differential expression genes co-expressed at different time points of deutonymph. (**C**) The statistics of pathway enrichment of all transcript mRNAs in different developmental time points of deutonymph.

A total of 3234 genes were expressed in development process of deutonymph in total. The differential expression genes (DEGs) (fold change ≥ 2.0 and *p* < 0.01) during different development time points were identified by comparing the expression level of transcripts at each time point with that at the 7 h time-point (14 h/7 h, 21 h/7 h, 28 h/7 h, and 35 h/7 h). Among these transcripts, 309 DEGs at 14 h compared that at the 7 h time-point, including 208 upregulated genes and 101 downregulated genes. 876 DEGs were identified in 21 h/7 h, including 540 genes were upregulated and 336 genes were downregulated. There were 2736 DEGs at 28 h compared that at the 7 h time-point, including 1616 upregulated genes and 1120 downregulated genes. There were 3432 DEGs at 35 h compared that at the 7 h time-point, including 1964 upregulated genes and 1468 downregulated genes (Figure 1A). A total of 79 upregulated and 42 downregulated genes were co-expressed in development process of deutonymph (Figure 1B). The KEGG analysis of DEGs showed that most DEGs belonged to the lysosome pathway (Table S1). These results indicated that more differentially expressed genes were involved in the molting process (Figure 1C).

### 3.3. Function Analysis of Differential Expression Genes in Development Process of Deutonymph

**Figure 2:**
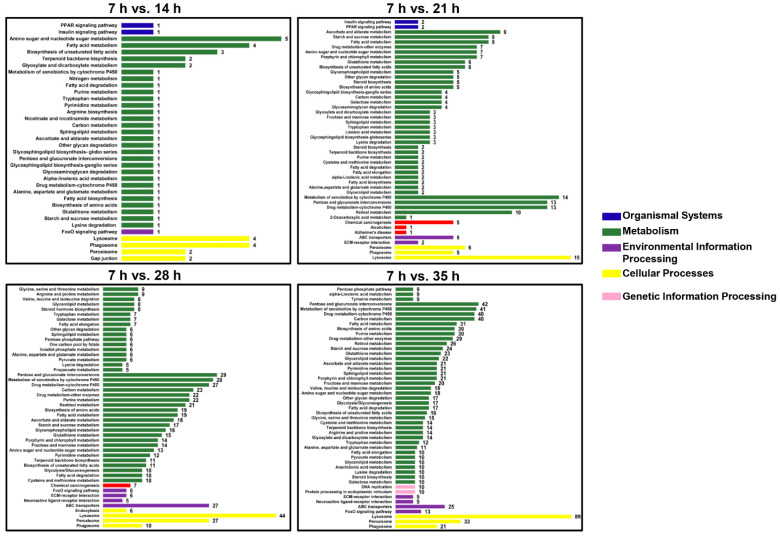
KEGG pathway classification for differential expression genes (DEGs) in different developmental time points (14 h vs. 7 h, 21 h vs. 7 h, 28 h vs. 7 h and 35 h vs. 7 h) of deutonymph in *T. urticae*.

To explore the function of the differential expression genes (DEGs) in the development process of deutonymph, the databases GO, KEGG, COG, NR, Pfam, eggNOG, and Swiss-Prot were used (Table 2). For the GO classification, the DEGs of four comparisons (14 h/7 h, 21 h/7 h, 28 h/7 h, and 35 h/7 h) in development process were divided into three classification: cellular component, molecular function, and biological process. GO enrichment analysis showed that most enriched GO terms were “membrane” in cellular component, “catalytic activity” in molecular function, and “metabolic process” in biological process in the different development time points compared with 7 h (Table S2). The KEGG analysis showed that most enriched classification was metabolism pathways, which contained “Amino sugar and nucleotide sugar metabolism”, “Fatty acid metabolism”, and “Biosynthesis of unsaturated fatty acids” in 7 h versus 14 h; “Ascorbate and aldarate metabolism”, “Starch and sucrose metabolism”, and “Fatty acid metabolism” in 7 h versus 21 h; “Pentose and glucuronate interconversions”, “Metabolism of xenobiotics by cytochrome P450”, and “Drug metabolism-cytochrome P450” in 7 h versus 28 h; “Pentose and glucuronate interconversions”, “Metabolism of xenobiotics by cytochrome P450”, and “Drug metabolism-cytochrome P450” in 7 h versus 35 h. Lysosome is the most enrichment pathway in cellular processes (Figure 2).

### 3.4. Expression Patters of mRNAs in Development Process of Deutonymph

**Figure 3:**
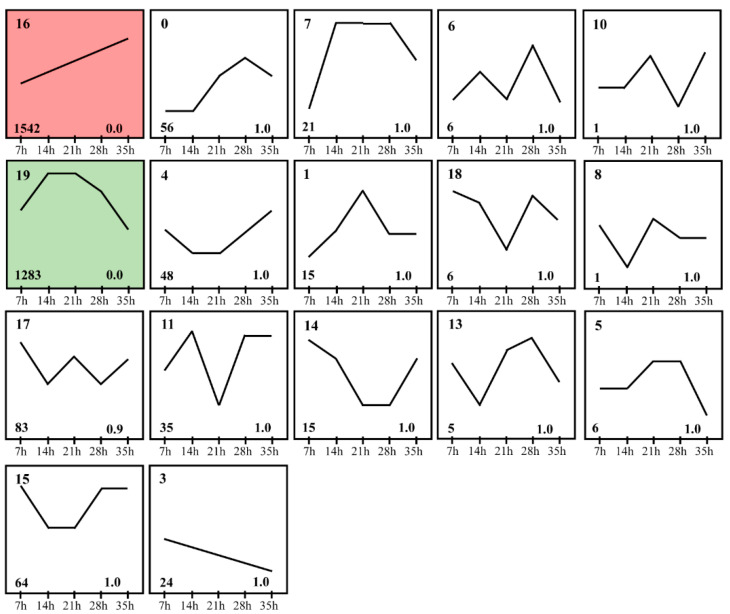
Differential expression clusters of differential expression genes in different developmental time points (7 h, 14 h, 21 h, 28 h and 35 h). Significant expression profiles (*p* < 0.05) of differential expression genes clustered via STEM software in different developmental time points. The numbers in the left upper part of boxes are profile serial numbers, those in left lower part are *p*-values, and those in the right lower part are numbers of transcripts contained in profiles.

**Figure 4:**
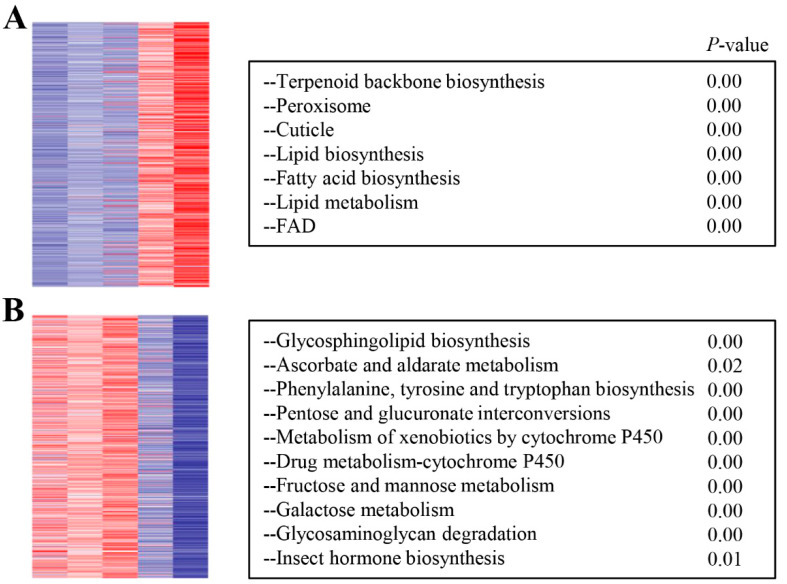
The heatmap of differential expression clusters and GO terms of profile 16 (**A**) and profile 19 (**B**) with corresponding enrichment *p*-values.

To investigate the expression dynamics of mRNA in development process of deutonymph, Short Time-series Expression Miner (STEM) was used to cluster transcripts based on their expression levels at different time points. The analysis result of STEM showed that just two expression clusters were assessed as significant expression profiles (*p* < 0.05) from the twenty model profiles. Of two expression profiles, 1542 and 1283 mRNAs were clustered into sixteen expression profiles and nineteen expression profiles, respectively. In the expression profile 16, the expression of these genes was increased constantly from 7 h to 35 h. In the expression profile 19, these genes were expressed highly at 14 h and 21 h, then the expression was constantly decreased from 21 h to 35 h (Figure 3). To identify the functions of these DEGs, the function analysis of these genes in two expression profiles was performed. In expression profile 16, these DEGs were involved in the cuticle, lipid biosynthesis, fatty acid biosynthesis, lipid metabolism, peroxisome, and terpenoid backbone biosynthesis (Figure 4A). In the expression profile 19, the DEGs were involved in insect hormone biosynthesis, metabolism of xenobiotics by cytochrome P450, and so on (Figure 4B and Table S3).

### 3.5. Expression Level Changes of Ecdysteroid and Juvenile Hormones Pathways Genes

**Figure 5:**
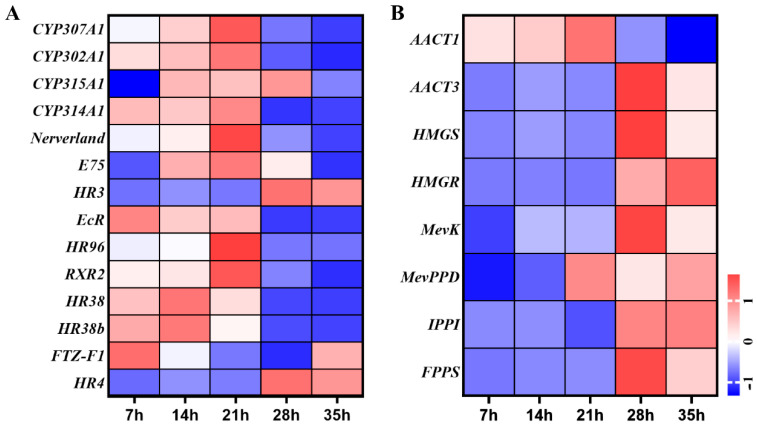
(**A**) Expression profiles of genes in ecdysteroids biosynthesis and signaling pathways of *T. urticae*. (**B**) Expression profiles of genes in juvenile hormone biosynthesis pathway of *T. urticae*. The color scales represent the values were normalized by Z-score ((value-mean value)/standard error) for each development time point.

Fourteen genes of ecdysteroid biosynthesis and signal pathway were involved in development process of deutonymph, including five ecdysteroid biosynthesis pathway genes and nine signal pathway genes. In the ecdysteroid biosynthesis genes, the expressions of *TuCYP307A1*, *TuCYP302A1*, *TuCYP315A1*, *TuCYP314A1*, and *TuNeverland* were gradually increased from 7 h to 21 h and then decreased until 35 h. In the signal pathway, the ecdysone receptor (*TuEcR*), *TuE75* and *TuHR38* were upregulated from 7 h to 14 h and downregulated from 21 h to 35 h, similar with the expression pattern of ecdysteroid biosynthesis genes. *TuRXR2* and *TuHR96* were significantly upregulated at 21 h, then downregulated at 28 h and 35 h. In contrast, the *TuHR3* and *TuHR4* were highly expressed from 28 h to 35 h (Figure 5A).

A total of 8 genes of sesquiterpenoid biosynthesis pathway were detected during the development process of deutonymph. Of these genes, the expression levels of *TuAACT1*, *TuHMGS*, *TuMevK*, *TuIPPI*, and *TuFPPS* were significantly high at 28 h and 35 h. The expression of *TuAACT3* were gradually increased from 7 h to 21 h and decreased from 28 h to 35 h. The expression level of *TuHMGR* was constantly increased from 7 h to 35 h. The expression of *TuMevPPD* was higher at 21 h and 35 h and decreased at 28 h (Figure 5B).

### 3.6. Expression Dynamics of Chitin Related Pathways Genes during Development Process of Deutonymph

**Figure 6:**
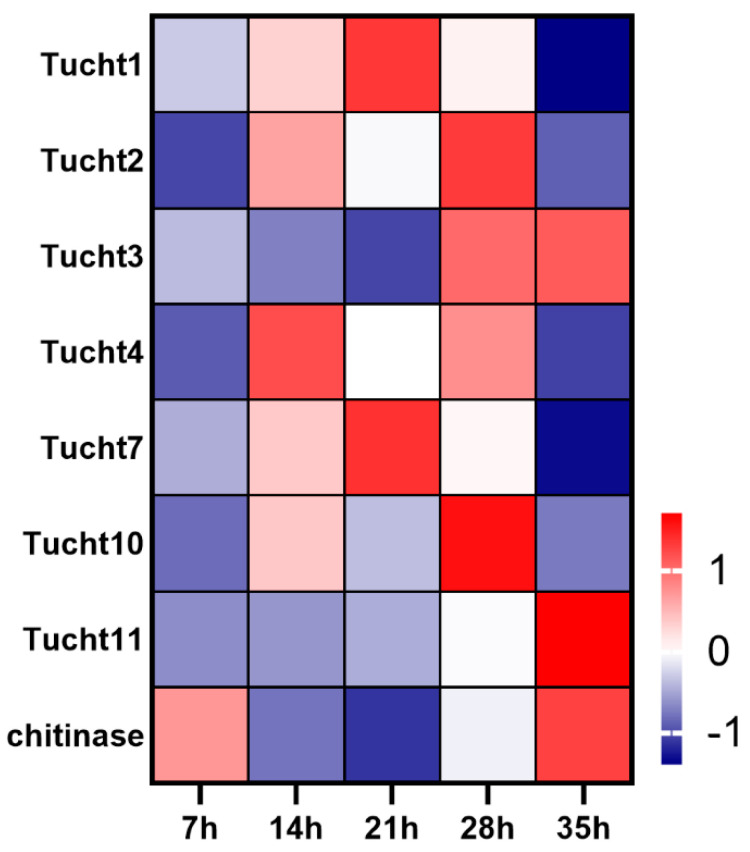
Expression profiles of chitinase genes during developmental process of deutonymph in *T. urticae*. The color scales represent the values were normalized by Z-score ((value-mean value)/standard error) for each development time point.

Thirteen chitinase were identified in the genome of *T. urticae*. Of these chitinase genes, 8 chitinase genes were expressed during developmental process of deutonymph. The expression levels of Tucht1 and Tucht7 were gradually increased from 7 h to 21 h and then decreased from 28 h to 35 h. Tucht2 and Tucht4 showed approximate “M” type expression pattern. Tucht10 was significantly highly expressed at 28 h. The expression level of Tucht3 and Tucht11 were constantly increased from 7 h to 35 h. Further, a horizontal transfer chitinase was highly expressed at 35 h (Figure 6).

### 3.7. Expression Pattern of Cuticle Protein Genes in Different Development Time Points

**Figure 7:**
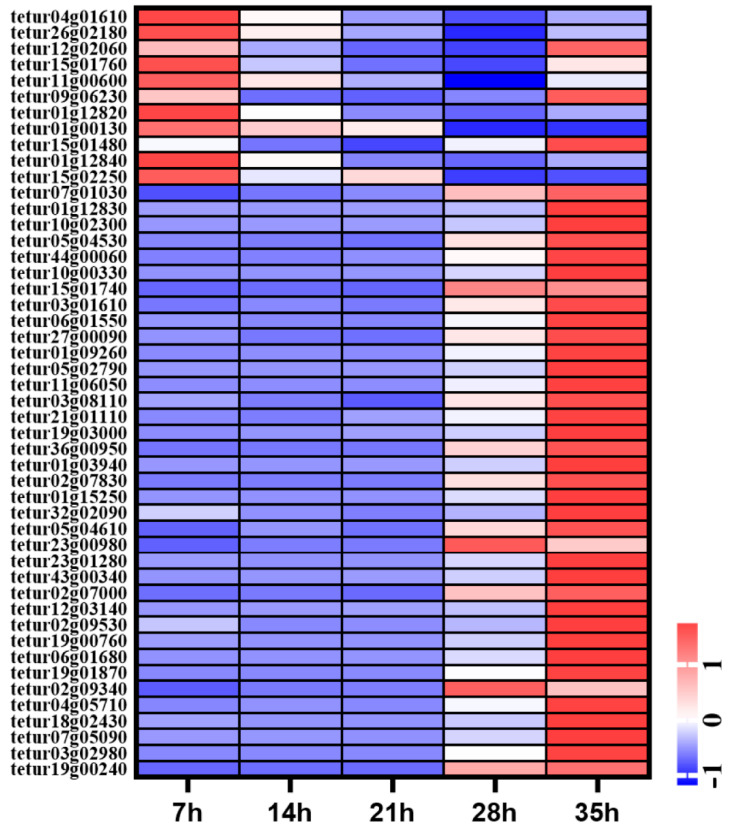
Expression profiles of CP genes in different time points of deutonymph in *T. urticae*. The color scales represent the values were normalized by Z-score ((value-mean value)/standard error) for each development time point.

A total of 59 cuticle protein related genes were identified in the genome of *T. urticae*, including 44 cuticle proteins (CPs) and 15 cuticle proteins analogous to peritrophins (CPAPs). A total of 44 cuticle proteins both belonged to the RR-2 family. Of these cuticle protein genes, 49 genes were expressed during development process. The expression levels of nine CPR genes were high at 7 h, then were decreased gradually from 14 h to 35 h. Three CPR genes were highly expressed at 7 h and 35 h. A total of 37 cuticle proteins including CPR and CPAP were significantly highly expressed at 35 h (Figure 7).

## 4. Discussion

Development is an important phylogenetic process including growth and molting [18]. In developmental process, arthropods undergo the forming and degradation of cuticle, internal tissues remodeling and apoptosis, and other physiological process [1]. The development process was regulated by multiple biological pathways such as RNA transport, insect hormone biosynthesis, and other in different insect species. In recent years, developmental transcriptome analysis in many insect species have been reported [19,20,21]. For the spider mites, they could quickly finish one lifecycle, which is shorter than insects. For example, the deutonymph of *T. urticae* could undergo both growth and molting processes within about two days. There is little study about the regulation dynamic of multiple biological pathways in the developmental processes of spider mites. To screen the regulate pathway networks in the development process of deutonymph in *T. urticae*, five different development time points from two different periods (growth period and molting period) of deutonymph (7 h, 14 h, 21 h, 28 h, and 35 h) in deutonymphs stage were collected for comparative transcriptome analysis. In total, 3234 differently expression genes were identified according to their expression levels during development process. Of these DGEs, more than 80% genes were differently expressed at molting process (genes expressed at 28 h and 35 h compared with 7 h). These results indicated that the genes of multiple biological pathways involved in molting process were more than that in growth process. The expression of 1283 DEGs were specifically increased in the growth process (from 7 h to 21 h), and many of these genes were related to immunity, digestion, and hormone regulation including insect hormone biosynthesis. Further, the expression of 1542 DEGs were persistently increased from 7 h to 35 h. Many were related to the cuticular related genes including cuticle protein gene and chitin genes. These results suggested that hormone- and cuticle-related genes have important function in the development process of deutonymph in *T. urticae*. The fat body is a key organ in metabolism, including energy storage and synthesis of carbohydrates, lipids and proteins. In the molting process, fatty acid biosynthesis was present, similar with the results of *Bemisia tabaci* [22], where these terms were closed related to fat accumulation. This fact suggests that fat synthesis was closely related to the molting process of *T. urticae*.

Ecdysteroid and juvenile hormone (JH) are two endocrine hormones that cooperatively regulate the growth and development in insects. The dynamic of two hormones defines the transition between growth and molting: when JH is absent, ecdysteroid initiates larval-pupal-adult metamorphosis, whereas the presence of JH ensures ecdysteroid drives the larval–larval molting during the middle larva instars [23]. In this present study of the spider mite *T. urticae*, the expression levels of five ecdysteroid biosynthesis pathway genes were increased from 7 h to 21 h in growth process and decreased from 28 h to 35 h in molting process, which was opposite to the expression patterns of seven juvenile hormone biosynthesis related genes. This result was consistent with the previous study about the expression patterns of ecdysteroid and juvenile hormone biosynthesis related genes in the molting processes of different development stages [14]. In the ecdysteroid signal pathway, the expression dynamics of most signal pathway related genes (*TuEcR*, *TuRXR2*, *TuHR38*, and *TuHR96*) were similar to the expression patterns of biosynthesis pathway genes, but the expression levels of *TuHR3* and *TuHR4* were increased in the molting process, opposite to the expression patterns of biosynthesis pathway genes. This expression pattern was similar with the expression of *PcHR3* in different time points of deutonymph in *P. citri* [24].

Cuticle is an effective barrier to help insects resist the dry environment and predation, and enhance athletic ability to increasing their survival probability. Cuticle proteins are the main components of cuticle. A total of 57 putative cuticular proteins were identified in *T. urticae* genome, including 42 CPR genes and 15 CPAP genes [17]. Of these CPR genes, 41 CPR belonged to the RR-2 class and one belonged to the RR-1 class. A total of 15 genes encoding two families of CPAPs were evaluated and divided into two families, CPAP1 and CPAP3, respectively. Of these CPs, all CPs but eight were highly expressed at the molting period. The expression levels of eight CPs were high after 7 h of molting in deutonymph. In insects, RR-2 class cuticle proteins were associated with rigid cuticles [11]. These results suggested that most of the cuticle proteins were related with the cuticle formation. However, the rigid cuticle also restricts the insect growth. Insects go through cyclical molting during development process, and the degradation of the old cuticular chitin is necessary in the molting process [25]. Insect chitinases are involved in the degradation of the old cuticle, and have unique developmental expression patterns in different insects [3,8,26]. In the present study, of *T. urticae*, eight chitinases were differentially expressed in the development process including a horizontal gene transfer chitinase from bacteria [27], and have different expression dynamics in the developmental process of deutonymph, suggesting that these chitinase may have different function in the development process.

## 5. Conclusions

Genes expression dynamic during different development time points of deutonymphal development process in *T. urticae* were investigated. The transcriptome sequencing aims to identify hormone- and cuticle-related genes that regulated the development process of spider mite. Moreover, this comprehensive transcriptome will provide a foundation for further research of development process in *T. urticae*.

## Figures and Tables

**Table 1:** Summary statistics of sequencing data for the transcriptomes of different development time points in *T. urticae*.

Samples	Replicates	Read Sum	Base Sum	GC (%)	Q30 (%)
7 h	1	19,966,666	5,964,221,328	39.22	93.38
	2	23,132,933	6,910,020,364	39.05	93.19
	3	19,239,246	5,753,690,284	39.16	93.16
14 h	1	21,402,946	6,397,745,756	38.88	92.81
	2	19,627,145	5,857,497,150	38.95	92.74
	3	21,981,138	6,588,408,854	37.49	92.34
21 h	1	23,280,649	6,901,943,666	38.76	92.51
	2	20,689,137	6,184,409,592	38.97	93.47
	3	19,498,291	5,808,116,624	38.88	92.93
28 h	1	20,130,351	6,010,236,656	38.96	92.86
	2	19,739,950	5,886,058,026	38.40	92.37
	3	19,485,760	5,825,531,426	38.31	92.72
35 h	1	20,756,485	6,203,250,222	39.17	93.26
	2	20,604,866	6,166,389,390	38.62	92.48
	3	20,504,881	6,130,799,544	38.43	93.16

Read Sum: pair-end reads in clean data. Base Sum: the total base numbers in clean data. GC: the percentage of G and C in clean data. Q30: nucleotides with a quality value above 30 in reads.

**Table 2:** Summary statistics of differential expression genes in different time points of deutonymph in *T. urticae*.

DEG Set	Total	Annotated	GO	KEGG	COG	NR	eggNOG	Swiss-Prot	KOG	Pfam
7 h vs. 14 h	242	213	442	56	64	211	130	91	92	128
7 h vs. 21 h	657	573	1270	267	237	569	397	298	342	380
7 h vs. 28 h	2169	1855	1005	835	732	1845	1332	992	1191	1279
7 h vs. 35 h	3075	2576	1421	1330	1030	2563	1871	1385	1676	1824

DEG: differential expression genes; GO: Gene Ontology; KEGG: Kyoto Encyclopedia of Genes and Genomes; NR: NCBI non-redundant protein sequences; eggNOG: Evolutionary Genealogy of Genes Non-supervised Orthologous Groups; Swiss-Prot: A manually annotated and reviewed protein sequence database; KOG: Eukaryotic Orthologous Groups. 7 h vs. 14 h:7 h versus 14 h; 7 h vs. 21 h:7 h versus 21 h; 7 h vs. 28 h:7 h versus 28 h; 7 h vs. 35 h:7 h versus 35 h.

## Data Availability

Data is contained within the article or Supplementary Materials.

## Supplementary Materials

The following are available online at https://www.mdpi.com/article/10.3390/insects12080736/s1, Table S1: The statistics of Kegg enrichment of differential expression genes in development process of deutonymph in *T. urticae*, Table S2: The GO terms statistics analysis of DEGs in different development time points of deutonymph in *T. urticae*, Table S3: The statistics of insect hormone and cuticle related genes in the expression profile 16 and profile 19.
